# Chlorotoxin—A Multimodal Imaging Platform for Targeting Glioma Tumors

**DOI:** 10.3390/toxins10120496

**Published:** 2018-11-26

**Authors:** Gadi Cohen, Scott R. Burks, Joseph A. Frank

**Affiliations:** 1 Frank Laboratory, Radiology and Imaging Sciences, Clinical Center, National Institutes of Health, Bethesda, MD 20892, USA; scott.burks@nih.gov; 2 National Institute of Biomedical Imaging and Bioengineering, National Institutes of Health, Bethesda, MD 20892, USA

**Keywords:** chlorotoxin, glioblastoma, imaging modalities

## Abstract

Chlorotoxin (CTX) is a 36-amino-acid disulfide-containing peptide derived from the venom of the scorpion *Leiurus quinquestriatus*. CTX alters physiology in numerous ways. It interacts with voltage gated chloride channels, Annexin-2, and matrix metalloproteinase-2 (MMP-2). CTX-based bioconjugates have been widely subjected to phase I/II clinical trials and have shown substantial promise. Many studies have demonstrated that CTX preferentially binds to neuroectodermal tumors, such as glioblastoma, without cross-reactivity to normal brain cells. With its ability to penetrate the blood-brain-barrier (BBB) and its tyrosine residue allows covalent conjugation with functional moieties, CTX is an attractive platform to explore development of diagnostic and therapeutic agents for gliomas. In this review, we outline CTX structure and its molecular targets, summarize molecular variations of CTX developed for glioma imaging, and discuss future trends and perspectives for CTX conjugates as a theranostic agent.

## 1. Chlorotoxin (CTX) as A Potential Targeting Agent for Glioblastoma

**Figure 1:**
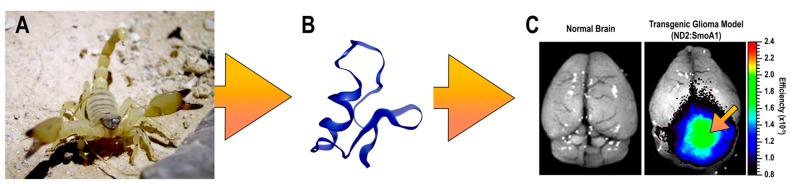
(**A**) The Israeli scorpion Leiurus quinquestriatu, from which its venom, chlorotoxin (CTX), is derived. (**B**) Three-dimensional structure of CTX reproduced from 1CHL PDB file [17], 1995, Biochemistry. (**C**) Visualization of IRDye 800CW-CTX targeting spontaneously develops medulloblastoma tumors in ND2:SmoA1 mice GBM. Left side: normal mouse brain. Right side: transgenic mouse model. Tumor location is marked using an orange arrow. Adapted with permission from Akcan et al. Reproduced from Reference [18], 2011, American Chemical Society.

Glioblastoma multiforme (GBM), represents the most common primary brain malignancy and is defined by its invasiveness and capacity for proliferation [1]. Although imaging techniques continue to advance, prognoses remain poor with a five-year survival rate of 5.5% [2,3]. Current standard therapies include surgical resection, followed by concurrent radiation with adjuvant chemotherapy [4]. Extensive surgical resection of GBMs is problematic as these tumors are highly invasive and extend into anatomical brain regions that are not amenable to surgery [5]. Preoperative planning now incorporates a variety of imaging modalities, including functional magnetic resonance imaging (MRI) and diffusion tensor imaging, computed tomography (CT), and MRI with direct stimulation during surgery, which have enabled more extensive resection capabilities that lessen the impacts on neurological function or quality of life [6]. Despite these advances, distinguishing normal brain from residual tumor tissues remains difficult [7]. Moreover, determining a patient’s glioblastoma subtype by immunohistochemistry remains a diagnostic challenge for the neuropathologist since hematoxylin and eosin staining of GBM can reveal a wide variety of pathologies that resemble other central nervous system lesions [8]. CTX is a 36 amino acids neurotoxin derived from the Israeli yellow scorpion’s venom, *Leiurus quinquestriatus* (Figure 1A). CTX has been purposed as a potential candidate for differentiating between molecular profiles and radiologic features of GBM, and could serve as a platform for developing novel noninvasive technique [9]. Molecular targets for CTX include voltage gated chloride channels [10,11,12], calcium-dependent phospholipid-binding protein Annexin-2 [13], and the inducible extracellular enzyme matrix metalloproteinase (MMP)-2 [12,13,14]. CTX has come to be an attractive platform for development of intraoperative imaging agents due to several structural advantages including; four disulfide linkages that impart a stable tertiary structure and a tyrosine residue that can be conjugate to a variety of imaging agents (Figure 1B) and its preferential binding to tumor cells (Figure 1C). CTX also binds glioblastoma tumor cells in a grade-related manner and does not cross-react with normal brain [15,16]. Given its unique structural and biological properties, CTX has the potential to be used as a targeting agent in a variety of applications used for diagnosing or treating GBM tumors.

## 2. CTX Structure and Molecular Targets

CTX is a 4 kD peptide consisting of 36 amino acids [19]. CTX is comprised of three short anti-parallel β-sheets tightly packed around an α-helix [17] and four disulphide linkages which contribute to a compact structure. The three-dimensional structure of CTX has been also determined by NMR spectroscopy (Figure 1B) [17,20]. CTX is named from its pharmacological effect of blocking small-conductance epithelial chloride channels [19]. Moreover, radio-receptor assay results were consistent with the chloride-conducting ion channel (ClC) family [10], and prolonged exposure to CTX stimulates intracellular internalization of these channels [12]. The discovery of specific CTX-sensitive glioma chloride currents in acute slices of human gliomas [21] highlight its potential use in glioma treatment. Even though gliomas present a wide degree of antigenic variability, they almost universally appear to over-express a CTX-sensitive chloride current [11,22]. These channels are absent or in low abundance in healthy tissues or in tumors of non-glial origin [23]. Interestingly, the expression level of these chloride channels appears to be correlated with the histopathological tumor grade despite the role of this channel type in the disease process remaining obscure. Experiments showed that submicromolar concentrations of CTX cannot block volume-regulated, Ca^2+^ activated and cystic fibrosis transmembrane conductance regulator chloride channels, suggesting CTX cannot be classified as a general chloride channel toxin [24]. Moreover, two binding sites for CTX were identified in vitro using glioma cell lines: a high affinity binding site with a Kd value of 4–9 nM and a low affinity one with a Kd in the 0.5–1 μM range. These results suggest more than one CTX-sensitive membrane receptor on the glioma cell surface [10] and may imply that the interaction between CTX and its target molecules are described by different binding affinities [14,25]. CTX has also been demonstrated to selectively and specifically act on matrix metalloproteinase (MMP)-2, which is expressed in glioma and other tumors, but is not present in normal brain tissues. It is part of the larger family of metalloproteases that have been associated with the enzymatic degradation of the extracellular matrix (ECM). Increased MMP-2 expression is related to the tissue invasion capacity of glioma cells. CTX can decrease the surface expression of MMP-2 and also inhibit its activity, thereby reducing overall invasion ability of glioma cells through compact extracellular spaces in normal brain tissue [14]. Although the molecular mechanisms of CTX in glioma remains elusive, there is a general agreement that CTX binds specifically to a membrane complex of chloride channel-3 (ClC-3) and MMP-2 [26], which may cause endocytosis of ClC-3/MMP-2 and a reduction of glioma invasiveness. 

In 2010, Kesavan et al. identified annexin A2 as a novel target molecule for TM-601 in human umbilical vein endothelial cell and multiple human tumor cell lines [27]. Annexin A2 is a calcium-dependent phospholipid binding protein present on the extracellular side of the plasma membrane of various tumor cells and endothelial cell types. It has many roles in cellular functions such as angiogenesis, apoptosis, cell migration, proliferation, invasion and cohesion. However, little is known about the inhibition of glioma invasion in the brain by CTX with regard to its annexin A2 binding capability. TM-601 was found to bind the surfaces of Panc-1 cells depending on the level of annexin A2 expression. [27]. Small interfering ribonucleic acid (siRNA) knockout of annexin A2 results in reduced binding of a technetium-99m-labelled-TM601 in cell lines expressing annexin A2 [28].

## 3. Molecular Imaging of Glioblastoma

Several recent developments in cancer diagnosis and therapy have come from animal venoms. They often exhibit specificity and selectivity for tumor proteins have become an invaluable framework to develop future anti-cancer treatments [29]. Molecular-imaging techniques have integrated venom-based moieties into numerous applications for tumor visualization and/or delivery of therapeutic anti-tumor agents [29,30]. Labelling these therapeutic agents with potential optical, magnetic resonance, or nuclear medicine imaging moieties allows for validation of the therapeutic profile in vivo along with directly determining the bio-distribution of the material [31,32]. Considering its molecular structure, CTX-based platforms can potentially be harnessed for drug development. Many applications have used CTX-based strategies for developing novel therapeutic and diagnostic techniques for detecting glioma [33,34], as shown in Table 1.

### 3.1. CTX-Based Nuclear Imaging

CTX has been used with positron emission tomography (PET) and single photon emission computed tomography (SPECT), for the development of new oncology-related contrast agents. Further, CTX can allow for the generation of “theranostic” agents that combine imaging and therapeutic agents in the same molecule [59]. These imaging modalities are attractive techniques to study the biodistribution and pharmacokinetics of novel targeted therapies and provide specific information about tumor physiology [32,60]. Radioactive iodine (^125^I and ^131^I) attached to CTX was the first compound translated to the clinic. This compound was designed to inhibit tumor growth as a radiotherapy agent as well as assess tumor volume and anatomical location [36,37]. Studies using ^125^I-labelled CTX have revealed glioma binding sites, for which CTX has both high and low affinities and could aid in identifying cancerous cells in biopsies from human glioma patients. ^131^I-CTX preferentially accumulated in tumor tissues following intravenous injection into immuno-deficient mice bearing human gliomas [10,35]. Despite promising preclinical results, this compound with the commercial name ^131^I–TM601 did not kill glioma cells in vitro when given as a single agent. Moreover, the radiochemical was detected in the stomach, kidneys, and thyroid of these animals. ^125^I-CTX was rapidly cleared through the urinary system and from all tissues except the stomach and thyroid [54]. As a result of this study ^131^I–TM-601 was further developed as a targeting agent for delivering therapeutic payloads. This compound entered clinical trials in adult patients with advanced-stage malignant GBMs and melanomas, and was subsequently used to investigate recurrent GBM [36,37,61]. A phase I clinical trial reported that a single intracranial dose of ^131^I-TM-601 was well tolerated and cleared from the body between 24 and 48 h [37]. As determined by the preclinical studies, ^131^I-TM-601 accumulated in the tumor periphery, while radiation doses in normal tissues and organs were not significant. Adverse effects were minimal and primarily related to the route of administration with no major toxicity or mortality due to ^131^I-TM-601 administration have been reported [37,38,42].

^131^I-labeled dendrimers conjugated to CTX has also been used as theranostic SPECT agents. This dendrimer-based platform sequentially conjugate polyethylene glycol (PEG), with CTX as a targeting agent and 3-(4′-hydroxyphenyl) propionic acid-OSu (HPAO), followed by ^131^I radiolabeling. The binding specificity of this construct was demonstrated in vitro on a C6 cancer cell line and in vivo on an MMP2-overexpressing glioma mouse model [39]. CTX was also used as a targeting agent for other theranostic platforms designed for SPECT imaging. A novel CTX-based polymeric nanoparticle radiolabeled with ^99m^Tc that contained two cytotoxic agents, alisertib and silver nanoparticles, has been developed as a theranostic agent [40]. The targeting ability of this compound was tested on a U87MG glioblastoma cell line and in vivo in U87MG-tumor bearing mice. Another advanced approach utilized CTX-like peptide radiolabeled with ^131^I, a recombinant CTX-like toxin isolated from Buthus martensii Karsch (BmK) to inhibited glioma cells development and invasion [33,34,35]. Nevertheless, more detailed study of the safety and availability of these CTX-conjugates should take into account serum and albumin binding, binding to vessel walls, lymphocyte binding, and uptake by macrophages when used systemically. Although, the potential use of CTX for cancer theranostics is broad, only small number of compounds will reach the clinic. Therefore, more effort is needs in developing novel CTX-based theranostics for the diagnosis and treatment of gliomas.

### 3.2. CTX-Based MRI/Optical Imaging

CTX has been used as a target delivery of magnetic resonance imaging (MRI) contrast agents or nanoprobes (NP) to tumor tissues [51]. MRI contrast was used to determine the extent of the tumor and for defining its precise localization within the brain. Gadolinium (Gd(III)) is frequently chelated by small molecules such diethylenetriamine pentaacetate (DTPA) for routine MRI contrast [62]. Gd(III) chelates tend to be rapidly cleared from circulation and do not possess intrinsic targeting capabilities [38]. An attempt to improve the targeting of Gd-based contrast agents was the development of poly-l-lysine dendrigraft containing Gd(III) [58,63]. This construct was composed of a l-lysine dendritic macromolecule conjugated to CTX either with Gd chelates or distyryl-substituted boradiazaindacene (BODIPY) fluorophore. The addition of a targeting function to the contrast agent provided a better uptake and enhanced retention time in tumor cells without apparent toxicity [58]. Longer circulation times and greater retention at tumor sites was also achieved by using iron oxide nanoparticles (IONP) [64,65]. Typical IONPs are comprised of solid iron oxide cores (usually magnetite, Fe_3_O_4_, or its oxidized form maghemite, γ-Fe_2_O_3_) with a biocompatible polymer coating [66]. Local interactions between iron and water protons accelerates proton dephasing to shorten transverse T2 relaxation times and enhance MRI contrast upon T2* imaging [67,68,69]. Likewise, fluorescent dyes can also be linked onto the surface of IONPs through the coated polymer allowing for multimodality investigation (i.e., MRI and optical imaging) of brain tumors [70].

CTX can be functionalized onto the surface of IONP pre-coated with PEG or a copolymer of PEG and chitosan [53,55,56,71,72]. IONPs have also been conjugated to CTX and Cy5.5 to create multimodal agents detectable using MRI and optical imaging [56]. Preferential targeting abilities of CTX-NP-Cy5.5 were determined via specific binding to glioma cells in vitro compared to NPs without CTX conjugation. In a follow-up study, IONPs were coated with PEG/chitosan and conjugated with CTX and Cy5.5. The CTX-Cy5.5 NP were efficiently internalized by tumors cells and inhibited invasion of C6 glioma cells compared to unconjugated IONPs (≈45%) [43,71]. This nontoxic compound proved to be permeable through the BBB allowing for a prolong detection by optical imaging and MRI of tumor cells in genetically engineered mice [51]. Real-time biological information to precisely detect small foci of cancer cells along with tumor margins could be achieved by optical imaging [51]. Nevertheless, this study had several weaknesses that made it difficult to translate to the clinic such as weak optical and MRI signals and poor photo-stability. Moreover, it was the only study we could find showing that Cy5.5-conjugated CTX-labelled glioma cells without affecting the BBB [43]. Disturbance in the BBB by tumors may depend on the tumor type and the stage of progression. CTX has been reported to cross the BBB and blood tumor barrier [33], diffusing deeply into the tumors, while other targeting agents, such as antibodies, were not able to cross the BBB [38,50].

Optical imaging detects light emitted from fluorophores attached to ligands that bind specific molecular targets. A suitable intraoperative CTX-based conjugated for pre-malignant lesions identification and improve visualization of tumor boundaries are near infrared (NIR) fluorescent moieties. NIR dyes are poorly absorbed by water or hemoglobin thereby limiting the amount of interference from auto-fluorescence and optimizes signal intensity [51]. One such application is the complex Cy5.5-CTX composed of CTX conjugated to Cy5.5 [31,47]. Cy5.5-CTX demonstrated specific binding toward tumor cells, transfer across the BBB, and enabled the detection of metastatic cancer foci comprised of a few hundred cells. CTX complex was modified by Akcan et al. [28], resulting in a mono-labeled peptide containing a single NIR fluorescent molecule without impacting the functionality or efficacy of CTX. Other NIR fluorophore molecules have been designed with the ability to paint tumors by using a CTX targeting strategy that included IRDye800CW or indocyanine green (ICG) CTX:800CW or BLZ-100. Specificity and functionality of the targeted agent, IRDye 800CW-CTX, were confirmed in cell-based assays and specifically targeted tumor tissue in ND2:SmoA1 mice, a transgenic model that spontaneously develops medulloblastoma tumors (Figure 1C) [44]. Surprisingly, blocking of IRDye 800CW-CTX binding was observed at 4 °C, a temperature at which internalization is slower, while at room temperature it was unsuccessfully blocked. ICG-CTX conjugated to BLZ-100 allowed for clear visualization of human glioma cells implanted in mouse brains, whereas normal tissue did not take up this agent [48]. In addition, BLZ-100 was translated into the clinic and it is currently undergoing evaluation in Phase I clinical trials in adult patients with glioma (NCT02234297). Furthermore, this probe has been clinically evaluated for adult skin cancer (NCT02097875), sarcoma (NCT024643320), and for pediatric patients with central nervous system tumors (NCT02462629) [73].

Other promising fluorescence-based imaging probes developed for tumors detection are quantum dots (QDs). QDs can provide images across large ranges of wavelengths and have high quantum yields [74]. Unlike organic dyes, which often suffer rapid photo-bleaching [75,76], QDs are composed of semiconducting metals (e.g., Cd, Zn, Se, In, P, and As) that are resistant to photo-bleaching [77]. Furthermore, the quantum properties of QDs are tunable and can provide excitation/emission wavelengths ranging from ultraviolet (UV) to NIR. The outer shell of the QD could be modified by organic layers including PEG polymers, phospholipids, and peptides [78], thus preventing nonspecific absorption of proteins to their surface, increase blood half-life, and reducing toxicity [79,80]. CTX conjugated with cadmium-free silver-indium-sulfide (Ag-In-S or AIS) chalcopyrite QDs was used in cellular imaging studies [47]. This CTX-conjugated QD-micelles exhibited specific internalization into U-87 brain tumor cells. Although QDs offer potentially valuable benefits in preclinical oncology, such as drug targeting and in vivo biomedical imaging, under certain conditions, they pose risks to human health and the environment, constituting a barrier for their translation to clinical use [81]. Semiconducting polymer dots have aroused extensive interests as a novel family of fluorescent probes for tumor targeting applications [54,61]. Unlike QDs, polymer-based dots were developed from biologically non-toxic materials, using deep-red photo-acceptors and visible-light photo-donors. Wu et al. [34] developed a polymer-blend dots conjugate (PBdot-CTX) that was capable of permeating through the BBB and specifically targets tumor tissue in the ND2:SmoA1 medulloblastoma mouse model. The PBdot-CTX conjugate characterized to have a mean size of 15 nm; was resistant to photo-bleaching, 15 times brighter than QDs, and stable in serum for over 72 h [45].

Lastly, a new class of nanoparticles, upconverting nanoparticles (UNCP), has recently been reported as functionalized fluorescent imaging agents. UNCP absorb low energy NIR (930 nm) wavelengths of light and “upconvert” to emit in the red visible spectrum (660 nm). This allows deep tissue penetration of excitation light and minimizes auto-fluorescence. Thus, the resulting signal-to-noise ratios are exceptionally high. In addition, these nanoprobes are soluble in aqueous solutions, are extremely photo stable, and fluoresce over long periods of time [82,83]. Rare-earth UNCP of polyethylenimine coated hexagonal-phase NaYF_4_:Yb, Er/Ce nanorods were functionalized with CTX and were shown to targeted C6 glioma xenografted tumors in vivo without appreciable signs of toxicity [46]. Recently CTX was conjugated onto lanthanide-ion doped NaGdF4 nanoparticles have been exploited as a new generation of MRI/optical probes [57]. Targeting ability of CTX-NaGdF_4_:Ho^3+^ NPs towards glioma cells was confirmed in vitro and in vivo using MRI and fluorescence imaging. Despite progress in recent years, experimental animal studies may never accurately predict the outcomes of human clinical trials. Moreover, CTX bioconjugates are unlikely to retain similar properties as those of CTX. Therefore, further study and development of novel CTX-modalities are likely to lead to more effective strategies for brain tumor control.

### 3.3. US/Optoacoustic Imaging of Glioma: Current Progress and Expectations

Optoacoustic imaging has been explored preclinically and is an area of particular interest. Acoustic emissions can result from the absorption of pulsed light energy, which occurs in accordance to the profile of endogenous tissue chromophores. The magnitude of acoustic emissions is proportional to the magnitude of optical energy deposited in tissue, which allows biological structures to be visualized with high optical contrast and acoustical resolution [84]. This modality is a high-resolution imaging tool used to assess binding efficiencies of appropriate contrast agents [85]. Commonly used acoustic imaging contrast agents are microbubbles (MBs) and nanobubbles (NBs) [86]. MBs were originally developed as diagnostic ultrasound contrast agents but have since been explored for targeted drug delivery by enhancing vascular permeability through cavitation that occurs when bubbles reside in ultrasound fields [87]. Modifications of bubble surfaces permit targeting of diseased tissues, reduced immunogenicity, and prolonged circulation lifetimes. Various bubble formulations are used for drug [88,89] or gene delivery [90]. To the best of our knowledge, research on developing CTX-targeted MBs have not been reported. Potentially, photoacoustic and/or ultrasound imaging could be used in conjunction with CTX-targeted MBs or NBs and be a valuable theranostic tool for gliomas.

By combining transcranial focused ultrasound (FUS) with MB infusions can transiently disrupt the BBB with high spatial precision and improves delivery chemotherapies into brain tumors [91,92]. Several studies have demonstrated enhanced central nervous system (CNS)-blood permeability [93,94] and increased local drug concentrations [95,96,97] following FUS exposure. Furthermore, additional therapeutic biologics, such genes and antibodies, have also been successfully delivered to the brain using FUS [98]. The BBB opening is transient, allowing the time window during which maximal chemotherapy doses could be delivered to tumors. Repeated use of FUS may permit planned high dose chemotherapy for CNS disease with minimal systemic toxicity [99,100]. The development of dual imaging/therapeutic molecules based on a FUS-induced BBB opening and CTX-functionalized nanoparticles could be the next breakthrough as a theranostic agent in glioma treatment. CTX-modified doxorubicin (DOX)-loaded liposomes [101], covalently linked CTX to liposomes encapsulating small interfering RNAs [49,50], or antisense oligo-nucleotides [52,61] could potentially be utilized as combined agent in GBM treatment management.

## 4. Conclusions

Overall, multiple applications have utilized CTX as a targeting domain over the past decade. CTX exhibits several properties advocating its use as an ideal platform for developing novel methods for imaging and therapy of glioma: (i) CTX’s compact structure enables its penetration across the BBB; (ii) it preferentially binds gliomas and other cancers arising from the neuroectoderm like melanoma, neuroblastoma, and medulloblastoma, without binding to normal tissue; (iii) CTX enables prolonged retention due to the ability to be internalized into tumor cells; (iv) several human trials have been conducted without reported toxicity or immunoreactivity; (v) convenient structure modification via a tyrosine residue to conjugate a variety of imaging or therapy agents without compromising its functionality; and (vi) demonstrated tumor binding capabilities via specific recognition of MMP-2, chloride channels, and Annexin-2 [16,38,43,55,70,71]. All these make CTX a novel and versatile theranostic compound that could be a key part of future glioma-targeted imaging and therapy.

## Figures and Tables

**Table 1:** Summary of various CTX-conjugate compounds.

CTX-Conjugate	Imaging Modality	References
^131^I/^125^I-CTX (^131^I-TM601)	SPECT Imaging	[10,35,36,37,38]
^131^I-BmK-CT	SPECT imaging	[39]
Ag/Ali-PNPs-CTX-^99m^Tc	SPECT imaging	[40]
^131^I-HPAO-PEG-dendrimers-CTX	SPECT imaging	[41,42]
Cy5.5-CTX	Optical Imaging	[43]
Mono-labeled CTX-Cy5.5	Optical Imaging	[18]
IRDye 800CW-CTX	Optical Imaging	[44]
Pdot-CTX	Optical Imaging	[45]
PEI-NaYF(4):Yb, Er/Ce-CTX	Optical Imaging	[46]
QD(Ag-In-S/ZnS)-CTX	Optical Imaging	[47]
ICG-CTX (BLZ100)	Optical Imaging	[48]
NP–AF647-(DNA or siRNA)-CTX	Optical Imaging	[49,50]
IONP-PEG-CTX	MRI	[51]
IONP-PEG-Chitosan-DNA-CTX	MRI	[52]
MFNP-SiNP–CTX	MR/Optical imaging	[53]
IONP-PEG-Chitosan-Cy5.5-CTX	MR/Optical imaging	[54]
IONP-PEG-CTX	MR/Optical imaging	[55,56]
NaGdF_4_-Ho^3+^-CTX	MR/Optical imaging	[57]
Gd-DTPA/BODIPY-dendrigraft poly-L-lysines-PEG-CTX	MR/Optical imaging	[58]

CTX: Chlorotoxin; Bmk-CT: Buthus martensii Karsch CTX like toxine; Ag/Ali-PNPs: Silver and alisertib polymeric nanoparticle; HPAO-PEG: 3-(4′-hydroxyphenyl)propionic acid-OSu-polyethylene glycol; PBdot: Polymer-blend dots conjugate; PEI-NaYF(4): Yb, Er/Ce: Polyethylenimine-coated hexagonal-phase NaYF(4):Yb, Er/Ce nanoparticles; QD: Quantum dots; ICG: Indocyanine green; NP: Nanoparticles; IONP: Iron oxide nanoparticles; MFNP-SiNP: Magnetite nanoparticle clusters in fluorescent silica nanoparticles; Magnetic resonance imaging (MRI); NaGdF_4_-Ho^3^: Lanthanide-ion doped NaGdF_4_ nanoparticles; Gd-DTPA: Gadolinium- diethylenetriamine pentaacetate; BODIPY: Distyryl-substituted boradiazaindacene.
